# Air pollution, lung function and mortality: survival and mediation analyses in UK Biobank

**DOI:** 10.1183/23120541.00093-2024

**Published:** 2024-04-29

**Authors:** Anna L. Guyatt, Yutong Samuel Cai, Dany Doiron, Martin D. Tobin, Anna L. Hansell

**Affiliations:** 1 Department of Population Health Sciences, University of Leicester, Leicester, UK; 2 Centre for Environmental Health and Sustainability, University of Leicester, Leicester, UK; 3 National Institute for Health and Care Research Health Protection Research Unit in Environmental Exposures and Health, University of Leicester, Leicester, UK; 4 National Institute for Health and Care Research Leicester Biomedical Research Centre, University Hospitals of Leicester NHS Trust, Research & Innovation, Leicester General Hospital, Leicester, UK; 5 Respiratory Epidemiology and Clinical Research Unit, Research Institute of the McGill University, Montréal, QC, Canada; 6 These authors are joint first authors

## Abstract

**Background:**

Air pollution is associated with lower lung function, and both are associated with premature mortality and cardiovascular disease (CVD). Evidence remains scarce on the potential mediating effect of impaired lung function on the association between air pollution and mortality or CVD.

**Methods:**

We used data from UK Biobank (n∼200 000 individuals) with 8-year follow-up to mortality and incident CVD. Exposures to particulate matter <10 µm (PM_10_), particulate matter <2.5 µm (PM_2.5_) and nitrogen dioxide (NO_2_) were assessed by land-use regression modelling. Lung function (forced expiratory volume in 1 s (FEV_1_), forced vital capacity (FVC) and the FEV_1_/FVC ratio) was measured between 2006 and 2010 and transformed to Global Lung Function Initiative (GLI) z-scores. Adjusted Cox proportional hazards and causal proportional hazards mediation analysis models were fitted, stratified by smoking status.

**Results:**

Lower FEV_1_ and FVC were associated with all-cause and CVD mortality, and incident CVD, with larger estimates in ever- than never-smokers (all-cause mortality hazard ratio per FEV_1_ GLI z-score decrease 1.29 (95% CI 1.24–1.34) for ever-smokers and 1.16 (95% CI 1.12–1.21) for never-smokers). Long-term exposure to PM_2.5_ or NO_2_ was associated with incident CVD, with similar effect sizes for ever- and never-smokers. Mediated proportions of the air pollution–all-cause mortality estimates driven by FEV_1_ were 18% (95% CI 2–33%) for PM_2.5_ and 27% (95% CI 3–51%) for NO_2_. Corresponding mediated proportions for incident CVD were 9% (95% CI 4–13%) for PM_2.5_ and 16% (95% CI 6–25%) for NO_2_.

**Conclusions:**

Lung function may mediate a modest proportion of associations between air pollution and mortality and CVD outcomes. Results likely reflect the extent of either shared mechanisms or direct effects relating to lower lung function caused by air pollution.

## Shareable abstract

**Adverse effects of air pollution on lower lung function (FEV_1_) potentially mediate 10–30% of the effects of PM_2.5_ or NO_2_ on mortality and incident cardiovascular disease** https://bit.ly/4bJmWgf

## Introduction

According to the Global Exposure Mortality Model, particulate matter <2.5 µm (PM_2.5_) was estimated to cause nearly 9 million deaths worldwide in 2015, the majority of which were from cardiovascular disease (CVD) [1]. Ambient air pollution is also an established risk factor for impaired respiratory health. Long-term exposure to particulate matter <10 µm (PM_10_), PM_2.5_ or nitrogen dioxide (NO_2_) has been associated with impaired lung function, and prevalence and incidence of COPD [2–4]. Air pollution has also been associated with increased risk of multimorbidity [5].

Impaired lung function (as measured by forced expiratory volume in 1 s (FEV_1_) and forced vital capacity (FVC)) is predictive of all-cause and cardiovascular (CVD) mortality in general population cohorts in the UK [6, 7]; such associations are also seen among lifelong non-smokers, as reported in UK Biobank previously [7]. Mendelian randomisation studies have suggested that confounding by smoking alone does not explain the relationship between lung function and mortality [8, 9]. Few studies, however, have reported the potential mediating role of impaired lung function on the associations between air pollution and mortality or CVD outcomes [10], in particular in subgroups that differ by smoking status. Previous studies have shown that the effect of air pollution on lung function may differ by smoking status [2]; effects of air pollution in ever-smokers have been hypothesised as being harder to detect, since smoking may already impair lung function *via* similar pathways to air pollution. Moreover, when studying the relationship between lung function and CVD, associations in smokers are likely to be residually confounded by smoking intensity [7].

We previously reported in a cross-sectional analysis that ambient air pollution was associated with lower levels of lung function in UK Biobank [2]. In this present study, we further investigate the extent to which associations between air pollution and mortality or incident CVD are potentially driven by the effect of air pollution on the spirometric measures of FEV_1_, FVC and FEV_1_/FVC. Three theories have been proposed to explain the relationship between lung function and CVD, including shared risk factors (*e.g.* confounding of the association), lung function representing the end-point of multiple early-life risk factors that affect CVD, and that reduced lung function (in our study, proposed as a result of air pollution exposure) might cause endothelial dysfunction and promote atherosclerosis [11]. Whilst gaseous pollutants may reach the alveoli and diffuse across the blood–air barrier, particulate matter may lodge in proximal airways of decreasing calibre, according to particle size [12]. Therefore, it is hypothesised that some inhaled pollutants may directly translocate to the bloodstream and exert local effects on the vasculature, whereas others may provoke a pulmonary inflammatory response with systemic consequences [13, 14]. In the largest mediation analysis of its kind, we estimated the proportions of the air pollution–outcome associations explained by the relationship between air pollution and lung function impairment.

## Methods

### Study populations

UK Biobank is a prospective cohort study of more than 500 000 participants (aged 40–69 years at baseline, 2006–2010), recruited from general practices across the UK [15]. On joining the study, participants attended a clinical interview with a nurse, completed questionnaires on their health and lifestyle, and performed spirometry. UK Biobank includes linkage to electronic healthcare records, as well as ambient air pollution concentration estimates at residence (supplementary table S1). All participants provided written consent, and ethical approval at the inception of the overall UK Biobank study was obtained from the North West Multi-Centre Research Ethical Committee and Patient Information Advisory Group. These specific analyses have been approved as part of UK Biobank project 648.

### Study outcomes

The main study outcomes in this analysis include mortality (all-cause and CVD) and incident CVD. Dates and causes of death from linked death registry data were used to define all-cause and CVD mortality. Death from CVD was defined in participants with a primary cause of death specifying an International Classification of Diseases, 10th Revision (ICD-10) code from the list described in supplementary table S2 and supplementary methods. Incident CVD (fatal and non-fatal events) was defined using the aforementioned death registry data and from Hospital Episode Statistics (HES) data. ICD-10 codes that defined fatal and non-fatal incident CVD events are also described in supplementary table S2. Participants with prevalent CVD events at the time of entering the study were excluded. The origin for the time axis was set to year of birth, with subjects entering analysis on the date of the baseline study visit. Censor dates were as described previously [16]: censor dates for mortality were 31 January 2016 in England and Wales, and 30 November 2015 in Scotland (and for participants whose location was unclear). For incident CVD (based on death registry data and HES data), censor dates were 31 March 2015 (England), 31 October 2015 (Scotland) and 29 February 2016 (Wales). Participants with hospital admissions from multiple nations were excluded (n=2772), as a censor date could not be confidently ascribed.

### Air pollution estimates

Land-use regression (LUR)-based estimates of PM_10_, PM_2.5_ and NO_2_ for year 2010 [17, 18] were linked to the geocoded residential address of each participant in UK Biobank. The LUR models used AirBase routine monitoring data with geospatial variables on road network, land use, population density and altitude. Models were originally developed for the Greater London area, with cross-validation in the study area of R^2^=77%, 88% and 87% for PM_10_, PM_2.5_ and NO_2_, respectively. Evaluation of transferability of the LUR models across the UK was assessed by comparing the modelled estimates with those from the UK's Automatic Urban and Rural Network monitoring data [19]. The R^2^ values fell with distance from study area, but whereas R^2^ values remained >0.5 for NO_2_, they were lower for particulate matter in northern England and Scotland, therefore particulate matter data >400 km from Greater London was not assigned to these areas (and they were excluded from analysis).

### Lung function measurements

Best measures of FEV_1_, FVC and their ratio (FEV_1_/FVC) underwent quality control as described previously [20]; briefly, the “best measure” per individual was defined as the highest measure from the “acceptable” blows for FEV_1_ and FVC. FEV_1_/FVC was derived from the selected FEV_1_ and FVC. Pre-bronchodilation lung function tests were performed using the Pneumotrac 6800 spirometer (Vitalograph, Maids Moreton, UK) by trained staff. Further outlier exclusions were undertaken by calculating per-trait z-scores, using the Global Lung Function Initiative (GLI) 2012 lung function equations, with any participant with an absolute GLI z-score value >5sd from the mean for any trait being excluded [21]. As explained previously, an individual's GLI “z-score” indicates their relative position (in terms of an sd (z) score) on a distribution for a given lung function trait in a population of non-smokers of a comparative age, sex and height [7]. Lung function GLI z-scores were used in the analysis, with hazard ratios (HRs) given for a 1-unit decrease in lung function GLI z-score (where a decrease in score indicates worse lung function).

### Statistical analysis

**FIGURE 1:**
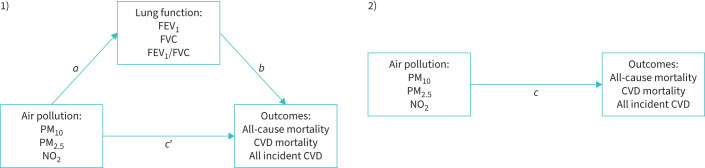
Directed acyclic graphs (DAGs) detailing the analyses in this paper. The aim of the paper is to understand the extent to which the relationship between air pollution and outcomes studied (all-cause mortality, cardiovascular disease (CVD) mortality and all incident CVD) may be mediated by lung function impairment (see DAG 1). *a* indicates the relationship between air pollution and lung function, which has already been studied in detail in Doiron *et al*. [2]. *b* indicates the relationship between lung function and the outcomes (see table 3; all-cause and CVD mortality have been previously studied in Gupta and Strachan [7], this analysis additionally studies fatal and non-fatal CVD events and extends analyses to smokers). The sum of *a*+*b* indicates the indirect effect of air pollution on the outcomes that passes *via* lung function. *c*′ indicates the direct effect of air pollution on the outcomes. The total effect of air pollution on the outcomes *c* is estimated in table 3. The mediated proportion is the indirect effect *a*+*b* divided by the total effect *c* and is given in table 4. PM_10_: particulate matter <10 µm; PM_2.5_: particulate matter <2.5 µm; NO_2_: nitrogen dioxide; FEV_1_: forced expiratory volume in 1 s; FVC: forced vital capacity.

We first performed survival analyses (figure 1) investigating: 1) lung function in relation to all-cause mortality, CVD mortality and incident CVD, and 2) the relationship between air pollution and the same outcomes. Smoking-stratified analyses and analyses in smokers adjusting for smoking intensity were conducted to obtain more accurate estimates in ever-smokers. Effect modification by anthropometric and sociodemographic factors (including occupation) and asthma was explored. Finally, we conducted mediation analyses to examine proportions of the air pollution–outcome associations that could potentially be explained by the relationship between air pollution and FEV_1._

Rates of all-cause and CVD mortality, and incident CVD, were expressed per 10 000 person-years, stratified by age group and calendar year. Rates were calculated for 2008–2015, because although data for 2006–2016 exist, there were only small numbers of events in 2016 (since the last date of linkage in England, where the majority of participants live, was in 2015) and relatively few participants were recruited in 2006–2007.

Survival analysis was performed using Cox proportional hazards models. The origin for the time axis was set to year of birth, with subjects entering analysis on the date of the baseline study visit. Proportional hazard assumptions were tested between each lung function GLI z-score and air pollution variable with study outcomes, by plotting –ln(−ln(survival)) *versus* ln(analysis time) curves, per quintile of exposure. Correlations between Schoenfeld residuals and log(time) were then assessed.

Survival analyses were stratified by “ever” *versus* “never” smoking status and adjusted for covariates (see below). 1) FEV_1_, FVC and FEV_1_/FVC were explored as predictors of mortality (all-cause and CVD) and incident CVD. Hazard ratios were expressed per 1-unit decrease in GLI z-score. 2) PM_10_, PM_2.5_ and NO_2_ were investigated as predictors of the same outcomes. Hazard ratios were expressed per interquartile range (IQR) increase in each pollutant.

The proportion of the associations between air pollution and the outcomes that might be driven by FEV_1_, FVC and FEV_1_/FVC was investigated in mediation analyses, first in all participants and then stratified by smoking status. Analyses were only conducted for pollutants with evidence of an association with the outcomes. The estimated mediated proportion was computed at the median of the lung function trait and the exposure effect was compared across the IQR. Cox regression was used to model the outcome and linear regression was used to model the mediator.

Survival and mediation analyses were adjusted for the following potential confounders: sex, height (cm), body mass index (kg·m^−2^), average household income before tax (dichotomised into <GBP 31 000 and ≥GBP 31 000), education level (categorised into “None”, “O Level, CSE or GCSE”, “A2, AS, NVQ, HNDC or Other” and “Degree”) and passive smoking (exposure to household smoke ≥1 h·week^−1^). In ever-smokers, results were further adjusted for smoking intensity, using “pack-years” (1 pack-year=20 cigarettes smoked per day per year).

Where a lung function or air pollution measure showed evidence of association with study outcomes, evidence of effect modification was assessed, using interaction terms, and then subgroup analysis, for: male *versus* female sex, age ≥65 *versus* <65 years, obesity (≥30 kg·m^−2^) *versus* non-obesity (<30 kg·m^−2^), “ever” *versus* “never” smoking status, income (≥GBP 31 000 *versus* <GBP 31 000), asthma *versus* no asthma and in those having a high-risk occupation for COPD [2, 22] *versus* those who did not (see supplementary table S3 for occupations considered). Covariates were as above.

Analysis was by complete-case analysis. Since many ever-smoker participants had missing data for “pack-years”, two sensitivity analyses were conducted, each omitting pack-years from the survival analyses for lung function and air pollution. First, the pack-years variable was omitted in ever-smokers with pack-years data. This analysis assessed sensitivity of the results to adjusting for smoking intensity. Next, pack-years was omitted again, but the sample of ever-smokers was expanded, this time also including ever-smokers without pack-years data. The latter analysis explored whether there was a selection effect when restricting to smokers with pack-years data, regardless of pack-years adjustment.

Stata version 16 (StataCorp, College Station, TX, USA) was used to undertake analyses, with the med4way package used to estimate mediated proportions in a survival model framework [23].

## Results

**FIGURE 2:**
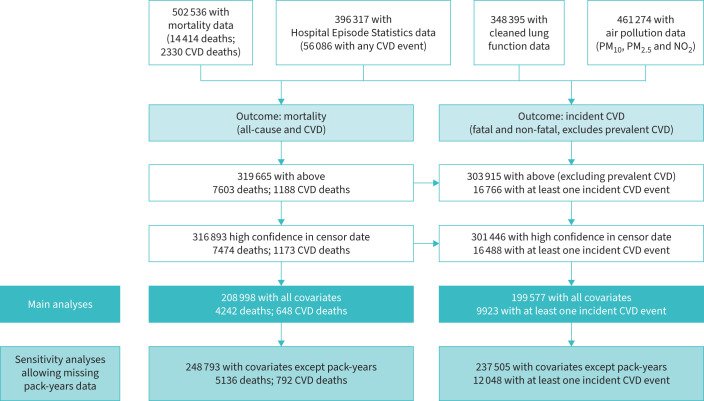
Flowchart detailing construction of the analysis sample in UK Biobank. CVD: cardiovascular disease.

A total of 208 998 individuals with complete covariate data (4242 deaths, of which 648 were CVD deaths) formed the “mortality analysis sample”. The “incident CVD analysis sample” (n=199 577, with 9923 incident CVD events) was a subset of these individuals which excluded those with prevalent CVD (see study flowchart in figure 2). Comparing the 316 893 individuals with incomplete covariate data to those in the mortality and incident CVD analysis samples, the latter two groups were more highly educated, had higher household incomes and were less likely to have ever smoked (table 1). Incidence rates of all-cause mortality, CVD mortality and incident CVD were fairly static over 2008–2015, with a slight increase over time in the oldest groups (supplementary figures S1–S3).

**TABLE 1:** Descriptive statistics for individuals with complete data on key exposures and outcomes *versus* those in two main analysis samples for mortality and incident cardiovascular disease (CVD)

**Factor**	**Level**	**Complete outcome data** **^#^**	**Mortality analysis sample**	**Level**	**CVD analysis (mortality** **and incidence) sample**
**Individuals**		316 893	208 998		199 577
**Outcome**	Alive	309 419 (97.6)	204 756 (98.0)	No CVD	189 654 (95.0)
	Died (other cause)	6301 (2.0)	3594 (1.7)	Incident CVD	9923 (5.0)
	Died (CVD)	1173 (0.4)	648 (0.3)		
**FEV_1_ (L)**		2.8±0.8 (n=316 893)	2.9±0.8 (n=208 998)		2.9±0.8 (n=199 577)
**FVC (L)**		3.7±1.0 (n=316 893)	3.7±1.0 (n=208 998)		3.7±1.0 (n=199 577)
**FEV_1_/FVC**		0.8±0.1 (n=316 893)	0.8±0.1 (n=208 998)		0.8±0.1 (n=199 577)
**COPD (GOLD stage)**	No COPD	272 468 (86.0)	182 930 (87.5)	No COPD	175 326 (87.8)
	Stage 1	20 610 (6.5)	12 850 (6.1)	Stage 1	12 247 (6.1)
	Stage 2	20 994 (6.6)	11 818 (5.7)	Stage 2	10 793 (5.4)
	Stage 3	2608 (0.8)	1287 (0.6)	Stage 3	1119 (0.6)
	Stage 4	213 (0.1)	113 (0.1)	Stage 4	92 (<1)
**PM_10_, 2010 (μg·m^−3^)**		16.0 (15.2–17.0) (n=316 893)	16.0 (15.2–17.0) (n=208 998)		16.0 (15.2–16.9) (n=199 577)
**PM_2.5_, 2010 (μg·m^−3^)**		9.9 (9.3–10.5) (n=316 893)	9.9 (9.2–10.5) (n=208 998)		9.9 (9.2–10.5) (n=199 577)
**NO_2_, 2010 (μg·m^−3^)**		25.9 (21.1–30.9) (n=316 893)	25.6 (21.0–30.7) (n=208 998)		25.6 (21.0–30.7) (n=199 577)
**Sex**	Female	176 405 (55.7)	116 341 (55.7)	Female	113 452 (56.8)
	Male	140 488 (44.3)	92 657 (44.3)	Male	86 125 (43.2)
**Age (years)^¶^**		56.3±8.1 (n=316 893)	55.9±8.0 (n=208 998)		55.6±8.0 (n=199 577)
**Age group^¶^**	<45 years	33 520 (10.6)	23 177 (11.1)	<45 years	23 018 (11.5)
	≥45 and <55 years	92 690 (29.2)	64 090 (30.7)	≥45 and <55 years	62 926 (31.5)
	≥55 and <65 years	133 931 (42.3)	87 247 (41.7)	≥55 and <65 years	82 801 (41.5)
	≥65 years	56 752 (17.9)	34 484 (16.5)	≥65 years	30 832 (15.4)
**BMI (kg·m^−2^)**		27.3±4.7 (n=316 623)	27.3±4.7 (n=208 998)		27.2±4.7 (n=199 577)
**BMI category^+^**	Normal	106 648 (33.7)	70 757 (33.9)	Normal	68 989 (34.6)
	Overweight	134 951 (42.6)	88 967 (42.6)	Overweight	84 822 (42.5)
	Obese	75 294 (23.8)	49 274 (23.6)	Obese	45 766 (22.9)
**Height (cm)**		168.4±9.2 (n=316 893)	168.6±9.1 (n=208 998)		168.5±9.1 (n=199 577)
**Education^§^**	None	48 954 (15.4)	26 351 (12.6)	None	23 867 (12.0)
	O Level/CSE/GCSE	54 517 (17.2)	35 019 (16.8)	O Level/CSE/GCSE	33 634 (16.9)
	A2/AS/NVQ/HNDC/Other	106 745 (33.7)	71 546 (34.2)	A2/AS/NVQ/HNDC/Other	68 345 (34.2)
	Degree	103 894 (32.8)	76 082 (36.4)	Degree	73 731 (36.9)
	Missing	2783 (0.9)			
**Income**	<GBP 31 000	125 953 (39.7)	92 645 (44.3)	<GBP 31 000	86 623 (43.4)
	≥GBP 31 000	148 228 (46.8)	116 353 (55.7)	≥GBP 31 000	112 954 (56.6)
	Missing	42 712 (13.5)			
**Smoking status**	Never-smoker	173 669 (54.8)	145 776 (69.7)	Never-smoker	141 055 (70.7)
	Ever-smoker	143 224 (45.2)	63 222 (30.3)	Ever-smoker	58 522 (29.3)
**Passive smoke exposure**	None	272 715 (86.1)	198 511 (95.0)	None	189 756 (95.1)
	Any	15 032 (4.7)	10 487 (5.0)	Any	9821 (4.9)
	Missing	29 146 (9.2)			
**Asthma**	Never had asthma	280 868 (88.6)	184 892 (88.5)	Never had asthma	176 542 (88.5)
	Ever had asthma	35 564 (11.2)	23 953 (11.5)	Ever had asthma	22 897 (11.5)
	Missing	461 (0.1)	153 (0.1)	Missing	138 (0.1)
**Occupation**	Non “at-risk” occupation	311 072 (98.2)	205 730 (98.4)	Non “at-risk” occupation	196 463 (98.4)
	“At-risk” occupation	5821 (1.8)	3268 (1.6)	“At-risk” occupation	3114 (1.6)

Data are presented as n, n (%), mean±sd or median (interquartile range). For continuous variables, numbers with non-missing data are given in brackets after the mean or median values (*e.g.* 2.9±0.8 (n=208 998)); for categorical variables, numbers missing are given as a separate category. FEV_1_: forced expiratory volume in 1 s; FVC: forced vital capacity; GOLD: Global Initiative for Chronic Obstructive Lung Disease; PM_10_: particulate matter <10 µm; PM_2.5_: particulate matter <2.5 µm; NO_2_: nitrogen dioxide; BMI: body mass index. ^#^: numbers of participants with incomplete covariate data; **^¶^**: at recruitment; ^+^: normal <25 kg·m^−2^, overweight 25–29.9 kg·m^−2^ and obese ≥30 kg·m^−2^; ^§^: the four categories correspond (approximately) to less than secondary education, secondary education, further education (usually 16–18 years) and degree level education (usually ≥18 years).

### Lung function, mortality and incident CVD

A decrease in FEV_1_ and FVC GLI z-scores was associated with increased all-cause mortality, CVD mortality and incident CVD (table 2). Point estimates for both mortality outcomes were larger in ever-smokers than never-smokers. For example, all-cause mortality HR per FEV_1_ GLI z-score decrease in ever-smokers 1.29 (95% CI 1.24–1.34) *versus* never-smokers 1.16 (95% CI 1.12–1.21); corresponding HR for CVD mortality in ever-smokers 1.43 (95% CI 1.31–1.57) *versus* never-smokers 1.31 (95% CI 1.18 1.45). Associations between FEV_1_ or FVC GLI z-scores and incident CVD were almost identical by smoking status. For all outcomes, associations with FEV_1_/FVC GLI z-score were found in ever-smokers only, and the relative magnitude of each association mirrored the results for FEV_1_ and FVC.

**TABLE 2:** Associations between lung function variables and mortality (all-cause and cardiovascular disease (CVD), n=208 998) and all incident CVD (n=199 577), stratified by smoking status

	**Individuals (n)**	**Outcomes (n)**	**Hazard ratio^#^ (95% CI)**		
			**FEV_1_**	**FVC**	**FEV_1_/FVC**
**All-cause mortality**					
Never-smokers	145 776	2267	1.16 (1.12–1.21)	1.17 (1.13–1.22)	1.05 (1.00–1.11)
Ever-smokers	63 222	1975	1.29 (1.24–1.34)	1.27 (1.22–1.33)	1.20 (1.14–1.25)
**CVD mortality**					
Never-smokers	145 776	286	1.31 (1.18–1.45)	1.38 (1.24–1.54)	1.01 (0.88–1.17)
Ever-smokers	63 222	362	1.43 (1.31–1.57)	1.42 (1.29–1.57)	1.29 (1.16–1.43)
**All incident CVD**					
Never-smokers	141 055	5743	1.12 (1.10–1.15)	1.14 (1.11–1.17)	1.01 (0.98–1.04)
Ever-smokers	58 522	4180	1.12 (1.09–1.15)	1.11 (1.08–1.14)	1.09 (1.05–1.13)

FEV_1_: forced expiratory volume in 1 s; FVC: forced vital capacity. ^#^: hazard ratios are per Global Lung Function Initiative z-score decrease for each lung function measure. Adjustments were as described in the Statistical analysis section of the Methods.

For all-cause mortality, there was evidence for a greater effect of impaired lung function in ever-smokers (HR for interaction (FEV_1_×ever-smoker) 1.10; p=0.001) and those from low-income households (HR for interaction (FEV_1_×higher income) 0.91; p=0.002). For incident CVD, effect sizes were slightly smaller in males (HR for interaction (FEV_1_×male sex) 0.93; p=1.20×10^−4^) (supplementary table S4).

### Air pollution, mortality and incident CVD

Hazard ratios are reported in terms of an IQR increase in air pollutants. Median (IQR) values for PM_10_, PM_2.5_ and NO_2_ were 9.9 (1.3), 16.0 (1.8) and 25.6 (9.7) μg·m^−3^, respectively.

For PM_2.5_ and NO_2_, there was evidence of association with all-cause mortality, and associations were similar in never-smokers (HR per IQR 1.04 (95% CI 0.99–1.09) for PM_2.5_ and 1.04 (95% CI 0.98–1.10) for NO_2_) and ever-smokers (HR per IQR 1.05 (95% CI 0.99–1.10) for PM_2.5_ and 1.05 (95% CI 0.99–1.11) for NO_2_) (table 3). Point estimates for associations between PM_2.5_ or NO_2_ and CVD mortality were higher in ever-smokers (HR per IQR 1.15 (95% CI 1.02–1.30) for PM_2.5_ and 1.11 (95% CI 0.97–1.27) for NO_2_) and close to the null in never-smokers. There was also evidence of association between PM_2.5_ or NO_2_ and incident CVD, with similar associations in never-smokers (HR per IQR 1.05 (95% CI 1.01–1.08) for PM_2.5_ and 1.05 (95% CI 1.01–1.08) for NO_2_) and ever-smokers (HR per IQR 1.05 (95% CI 1.01–1.09) for PM_2.5_ and 1.04 (95% CI 1.00–1.09) for NO_2_). PM_10_ was not associated with either mortality or incident CVD outcomes.

**TABLE 3:** Associations between air pollution variables and mortality (all-cause and cardiovascular disease (CVD), n=208 998) and all incident CVD (n=199 577), stratified by smoking status

	**Individuals (n)**	**Outcomes (n)**	**Hazard ratio^#^ (95% CI)**		
			**PM_10_**	**PM_2.5_**	**NO_2_**
**All-cause mortality**					
Never-smokers	145 776	2267	0.98 (0.94–1.02)	1.04 (0.99–1.09)	1.04 (0.98–1.10)
Ever-smokers	63 222	1975	0.97 (0.93–1.01)	1.05 (0.99–1.10)	1.05 (0.99–1.11)
**CVD mortality**					
Never-smokers	145 776	286	0.93 (0.83–1.04)	1.01 (0.87–1.16)	0.99 (0.85–1.16)
Ever-smokers	63 222	362	1.02 (0.93–1.13)	1.15 (1.02–1.30)	1.11 (0.97–1.27)
**All incident CVD**					
Never-smokers	141 055	5743	1.01 (0.99–1.04)	1.05 (1.01–1.08)	1.05 (1.01–1.08)
Ever-smokers	58 522	4180	1.00 (0.97–1.02)	1.05 (1.01–1.09)	1.04 (1.00–1.09)

PM_10_: particulate matter <10 µm; PM_2.5_: particulate matter <2.5 µm; NO_2_: nitrogen dioxide. ^#^: hazard ratios are per interquartile range increase for the air pollution measure (see table 1). Adjustments were as described in the Statistical analysis section of the Methods.

There was no formal evidence of interaction between PM_2.5_ or NO_2_ and any of the covariates studied (plus asthma and occupation) in relation to study outcomes (all p_interaction_>0.1) (supplementary table S5).

### Testing proportional hazard model assumptions

Proportional hazard assumptions were tested for univariate Cox regression models between quintiles of each lung function and air pollution variable with the mortality and CVD incidence outcomes (supplementary figures S4–S6). There was some evidence of departure from the proportional hazards assumption for the CVD mortality analyses for FEV_1_ (p=0.031) and FEV_1_/FVC (p=0.001) GLI z-scores (supplementary figure S5).

### Sensitivity analyses

In the main analysis, when analysing smokers, we restricted to smokers with pack-years data available (n=63 222), and adjusted the association between lung function and mortality and CVD incidence outcomes for pack-years. However, many ever-smokers (n=39 975) had missing data for pack-years. Therefore, as sensitivity analyses (supplementary table S6), we performed two analyses without pack-years data, in 1) the same 63 222 individuals analysed in the main analysis and 2) in a larger sample of 103 197 smokers, regardless of whether or not they had pack-years data available. Point estimates were consistently larger in both sensitivity analyses where pack-years was not included, suggesting that controlling for smoking intensity was important, and drove the difference in the results in the main and sensitivity analyses (as opposed to a selection effect whereby those with pack-years data available differed greatly from those without it). A similar pattern of results (albeit with less pronounced differences) was seen for the air pollution analyses (supplementary table S7).

### Mediation analyses

Mediation analyses were only conducted for the associations between air pollution and all-cause mortality and incident CVD, as there was less consistent evidence of an association between air pollution and CVD mortality, as seen in table 3.

There was evidence that associations of PM_2.5_ or NO_2_ with all-cause mortality could be partially mediated *via* FEV_1_ (estimated proportion mediated 0.18 (95% CI 0.02–0.33) for PM_2.5_ and 0.27 (95% CI 0.03–0.51) for NO_2_) (table 4). Whilst smoking-stratified analyses may have been underpowered, point estimates were similar in magnitude for PM_2.5_, but larger in never-smokers for NO_2_ (although confidence intervals overlapped for estimates in ever- and never-smokers). For incident CVD, point estimates were larger for NO_2_ (0.16 (95% CI 0.06–0.25)), although confidence intervals overlapped with those of PM_2.5_ (0.09 (95% CI 0.04–0.13)). Differences in effect sizes by smoking status were not pronounced.

**TABLE 4:** Estimated proportions of associations between particulate matter <2.5 µm (PM_2.5_) and nitrogen dioxide (NO_2_)^#^ and all-cause mortality and incident cardiovascular disease (CVD), mediated by forced expiratory volume in 1 s (FEV_1_)

	**Coefficient^¶^** **(95% CI)**	
	**PM_2.5_**	**NO_2_**
**All-cause mortality**		
All	0.18 (0.02–0.33)	0.27 (0.03–0.51)
Never-smokers	0.16 (−0.05–0.38)	0.28 (−0.12–0.67)
Ever-smokers	0.15 (−0.05–0.35)	0.19 (−0.04–0.43)
**All incident CVD**		
All	0.09 (0.04–0.13)	0.16 (0.06–0.25)
Never-smokers	0.11 (0.03–0.19)	0.20 (0.04–0.35)
Ever-smokers	0.06 (0.01–0.11)	0.10 (0.00–0.19)

^#^: analyses not run for particulate matter <10 µm, since there was no strong evidence of association between this pollutant and mortality or incident CVD; ^¶^: coefficients are estimate proportions of the effect of the pollutant on the outcome mediated *via* FEV_1_. Results were produced using the med4way Stata package, specifying the lower and upper quartiles of the air pollutants as the referent (a0) and actual (a1) levels of the exposure (a0), the median of FEV_1_ as the level of the mediator at which the four-way decomposition is computed (m), “cox” as the form of regression model for the outcome (yreg) and “linear” as the form of the regression model for the mediator (mreg). Confounders (c) were fixed at female sex, income <GBP 31 000, no qualifications, no passive smoke exposure, and at the means of height, body mass index and pack-years smoked.

Mediation analyses studying FVC and FEV_1_/FVC as mediators are presented in supplementary table S8. Generally, larger point estimates were observed for all-cause mortality compared with incident CVD, and the magnitude of the proportion of effect mediated was smaller for FVC and FEV_1_/FVC compared with FEV_1_. Confidence intervals for analyses stratified by smoking were broad, but for incident CVD, the possibility of mediation of pollutant–outcome exposures by FVC was stronger in never-smokers, whereas for FEV_1_/FVC it was stronger in ever-smokers.

## Discussion

In previous analyses of UK Biobank, we found that air pollution was associated with impaired lung function [2]. Whilst the relationship between mortality and lung function has been studied previously in lifelong non-smokers in UK Biobank [5], in the current study, we additionally studied smokers. We found that lower levels of FEV_1_ and FVC were associated with all-cause mortality, CVD mortality and incident CVD regardless of smoking status, whereas associations with FEV_1_/FVC were mainly apparent in ever-smokers. We also found associations between air pollutants and mortality or incident CVD that were most prominent for NO_2_ and PM_2.5_, with no strong associations found for PM_10_. Mediation analyses suggested that FEV_1_ could mediate 18% of the association between exposure to PM_2.5_ and all-cause mortality, and 27% of the association with NO_2_. Estimated mediated proportions by FEV_1_ for the associations of the same pollutants with incident CVD were smaller (9% and 16%, respectively).

Our observed associations between PM_2.5_ and NO_2_ and all-cause mortality, as well as incident CVD, were comparable to those found in the ELAPSE (Effects of Low-level Air Pollution: a Study in Europe) project [24, 25]. The main biological mechanisms for the effects of PM_2.5_ or NO_2_ on morbidity and mortality proposed include promotion of oxidative stress and local pulmonary inflammation, which subsequently leads to subclinical systemic inflammation. Pathways involving increased oxidative stress and inflammation may drive CVD risk, *via* promoting atherosclerosis, endothelial dysfunction, a prothrombotic state, and alterations to the electrophysiology of the heart and blood pressure regulation [26]. Ultrafine particulates (*e.g.* particulate matter <0.1 µm (PM_0.1_)) and noxious gases (*e.g.* NO_2_) may also translocate/diffuse into the bloodstream and exert a direct effect on the heart and vasculature [12]. The theory that reduced lung function leads to an increase in systemic inflammation and then to atheroma formation is one of the three theories proposed by McAllister and Newby [11] to explain a relationship between lung function and CVD, with other hypotheses being that the association is confounded or observed because lung function is the end-point of multiple adverse fetal and early-life factors which affect CVD risk.

Our mediation analysis suggested that, in addition to the pathways described above, lung function impairment may partially explain the associations between long-term exposure to ambient air pollution and mortality or incident CVD. For FEV_1_ and FVC, this result was more prominent in never-smokers; for FEV_1_/FVC, the result was more prominent in ever-smokers. Our results in all participants are in broad agreement with a previous mediation analysis from the SALIA (Study on the influence of Air pollution on Lung function, Inflammation and Ageing) cohort of 2527 elderly women in Germany [10]. Although the outcome assessed in the SALIA study was cardiopulmonary mortality, the confidence intervals of the proportion of the associations of NO_2_ with mortality and incident CVD mediated by FEV_1_ in our analysis are consistent with the SALIA estimate (in SALIA, 16.5% of the NO_2_–cardiopulmonary mortality association was estimated to be mediated *via* FEV_1_). Both studies have found that FEV_1_ mediated less of the association between PM_2.5_ and the outcomes studied, compared with analyses of NO_2_.

Oxidative stress and inflammation have also been suggested as key drivers of damage to the lung parenchyma, the physiological repair mechanisms and accelerated lung ageing [27]. The mechanisms of action for NO_2_ are less well characterised, but may also relate to oxidative stress [26], or concentrations may be a marker of ultrafine particulate matter (both are generated by combustion [28]), which may cause more pulmonary inflammation (which may initiate an acute-phase inflammatory response) and lodge for longer in the lung [29].

Our analyses demonstrated differing magnitudes of associations between lung function and mortality/CVD in ever- and never-smokers. We found that adjusting for smoking intensity (pack-years) in smokers attenuated effect sizes towards those observed in never-smokers. This did not appear to be due to a selection effect of those with non-missing data for smoking intensity. The persistent larger effect size of lung function–mortality estimates in smokers may be due to residual confounding as a result of measurement error in smoking intensity. We also observed higher effect sizes for lung function on all-cause mortality in those with lower household incomes and in males; a residual confounding effect by smoking intensity may explain these findings.

Further, our results for incident CVD suggest that up to 10–20% of the impact of air pollution may be mediated by lung function; therefore, at least 80% occurs through either a different mechanism or direct effects on the cardiovascular system.

To the best of our knowledge, this is the largest mediation analysis exploring the role of FEV_1_ in driving associations between air pollution, all-cause mortality and incident CVD, and includes a more diverse population (in terms of age and sex) than studied previously [10]. We were able to stratify by smoking status and control for smoking intensity in analyses of smokers. The large sample size is likely to have helped detection of a potential mediating effect of FEV_1_ on PM_2.5_–mortality and –CVD outcome associations; this more modest effect size for PM_2.5_ may explain why it was not detected in a previous, smaller study [10].

There are a number of limitations in this work. Air pollution model agreement with ground-level monitoring has been shown to be only moderate; if such error is random, this may bias point estimates towards the null. Air pollution estimates were modelled for 2010, potentially up to 4 years after spirometry measures were taken (2006–2010), meaning that the mediator measurement could precede the exposure measurement for some participants. However, there is evidence that air pollution measures are likely to have been relatively stable in the UK over these years [2, 30]. Despite the larger sample size, the follow-up period was shorter than other studies, which along with the strict definition of requiring an ICD-10 code as the primary cause, may have reduced the power of the CVD mortality analyses. Moreover, the analyses of CVD mortality and incident CVD may be vulnerable to bias from competing risks. Importantly, it is recognised that the relationship between lung function and CVD may not be causal [11]. Similarly, in this observational work, we cannot conclude causation from our mediation analyses, and differences in results by lung function traits, outcomes, pollutants and smoking status highlight the need to perform further work to interrogate any potential causal mechanisms. This may require even more complex modelling of additional environmental and non-environmental confounders, such as other cardiovascular risk factors (diabetes and blood pressure) and pollutants (*e.g.* ultrafine particulate matter), and consideration of mediation–outcome confounders (*e.g.* if systemic oxidative stress confounds the lung function–CVD relationship, rather than mediates it). Given the correlation between pollutants, which is particularly pronounced for NO_2_ and PM_2.5_, future analyses taking into account the dependencies between these variables would be important. Finally, we performed complete-case analysis, which will have limited the power of our analyses and may have led to an underrepresentation of participants from more disadvantaged socioeconomic backgrounds, which may in turn introduce the possibility of collider bias [31].

In conclusion, this study suggests that the effects of PM_2.5_ or NO_2_ on lung function may account for between 10% and 30% of the association between these pollutants and all-cause mortality and incident CVD. Future work is required in order to understand whether these associations are causal.

## Supplementary material

10.1183/23120541.00093-2024.Supp1 **Please note:** supplementary material is not edited by the Editorial Office, and is uploaded as it has been supplied by the author.
Supplementary material 00093-2024.SUPPLEMENT
Supplementary material 00093-2024.SUPPLEMENT
